# The challenge of consolation: nurses’ experiences with spiritual and existential care for the dying-a phenomenological hermeneutical study

**DOI:** 10.1186/s12912-015-0114-6

**Published:** 2015-11-24

**Authors:** Kirsten Anne Tornøe, Lars Johan Danbolt, Kari Kvigne, Venke Sørlie

**Affiliations:** Lovisenberg Diaconal University College, Lovisenberg gt. 15B 0456, Oslo, Norway; MF, Norwegian School of Theology, Gydas vei 4, Majorstuen 0302, P.O. Box 5144, Oslo, Norway; Religionspsykologisk Senter (Center for the Psychology of Religion) Innlandet Hospital, P.O. Box 68 2312, Ottestad, Norway; Department of nursing, Faculty of Public Health, Hedmark University College, P.O. Box 400 2418, Elverum, Norway; Department of nursing Nesna University College, Nesna, 8700 Norway

**Keywords:** Nurses’ challenges, Spiritual, Existential, Care, Dying patients, Hospitals, Phenomenological hermeneutical

## Abstract

**Background:**

A majority of people in Western Europe and the USA die in hospitals. Spiritual and existential care is seen to be an integral component of holistic, compassionate and comprehensive palliative care. Yet, several studies show that many nurses are anxious and uncertain about engaging in spiritual and existential care for the dying. The aim of this study is to describe nurses’ experiences with spiritual and existential care for dying patients in a general hospital.

**Methods:**

Individual narrative interviews were conducted with nurses in a medical and oncological ward. Data were analyzed using a phenomenological hermeneutical method.

**Results:**

The nurses felt that it was challenging to uncover dying patients’ spiritual and existential suffering, because it usually emerged as elusive entanglements of physical, emotional, relational, spiritual and existential pain. The nurses’ spiritual and existential care interventions were aimed at facilitating a peaceful and harmonious death. The nurses strove to help patients accept dying, settle practical affairs and achieve reconciliation with their past, their loved ones and with God. The nurses experienced that they had been able to convey consolation when they had managed to help patients to find peace and reconciliation in the final stages of dying. This was experienced as rewarding and fulfilling. The nurses experienced that it was emotionally challenging to be unable to relieve dying patients’ spiritual and existential anguish, because it activated feelings of professional helplessness and shortcomings.

**Conclusions:**

Although spiritual and existential suffering at the end of life cannot be totally alleviated, nurses may ease some of the existential and spiritual loneliness of dying by standing with their patients in their suffering. Further research (qualitative as well as quantitative) is needed to uncover how nurses provide spiritual and existential care for dying patients in everyday practice. Such research is an important and valuable knowledge supplement to theoretical studies in this field.

## Background

The evidence on death and dying in Western Europe and the USA suggests that a majority of people die in hospitals [1]. In spite of this, cure oriented hospital environments tend to focus on the physical aspects of illness, even though clinical research and experience shows that dying patients are confronted with complex and unique challenges that threaten their physical, emotional, and spiritual integrity and wellbeing [1–5]. In times of illness, what might loosely be called spiritual, meaning, and identity issues may come to the fore, even when religion and spirituality have not previously been of significance [6]. Drawing on Gibson et al. [7], Swinton and Pattison [6] maintain that the therapeutic focus on patients’ spiritual and existential issues has been shown to be preventative against depression, loss of self-value and the desire for suicide among people with terminal illness.

When the brutality of illness outstrips the power of medical technology, part of the fallout lands squarely on front–line clinicians [8]. As the largest professional group, registered nurses play an important role in hospital care for the dying [1]. Boston et al. [9], point out that dying patients frequently experience severe spiritual and existential anguish which according to Bruce et al. [10], can be described as a condition where morbid suffering may include concerns related to hopelessness, futility, meaninglessness, disappointment, remorse, death anxiety and a disruption of personal identity. Dying patients’ spiritual and existential suffering may have a profound and devastating impact on the wellbeing of family members because it prompts their awareness of loss, which may trigger grief, depression and anxiety [11, 12]. In spite of this, health care professionals tend to overlook family needs and family members are often referred to as “hidden patients” [11]. It is therefore crucial that nurses are able to discern spiritual and existential distress and its effects on overall family health; and that they are able to integrate a family perspective in their spiritual care interventions [11].

Kisvetrova et al. [13] point out that the purpose of spiritual and existential support is to alleviating dying patients death anxiety and distress, and that spiritual and existential care interventions may include religious as well as spiritual and existential aspects. According to them, spiritual and existential care interventions involve conveying empathy, active listening, being present with patients, helping patients to accept their thoughts and feelings around death and dying, showing respect and supporting patients’ dignity. They also emphasize the importance of creating a compassionate and caring environment to bring hope, help patients to deal with the reality of death and to support their spiritual well being in the terminal stage of life [13].

According to Balboni et al. [14], spiritual care is associated with key patient outcomes, including patient quality of life, satisfaction with hospital care, increased hospice use, and decreased aggressive medical interventions and medical costs. It is therefore vital that hospital nurses are able to provide spiritual and existential care for dying patients. However, several studies reveal that this is still a major challenge for many. While maintaining curative responsibilities for patients with life threatening illnesses, nurses must also be able to address the needs of the dying as part of their daily work [1, 2, 5, 15].

In light of the growing body of palliative care research, spiritual and existential care for the dying is seen to be an integral component of holistic, compassionate and comprehensive palliative care [4, 16–19]. Nevertheless, research also reveals that spiritual and existential care is frequently overlooked in palliative care [16]. Patients with advanced illnesses report that their medical caregivers infrequently provide spiritual care [14]. According to Udo [20], several studies show that many patients are dissatisfied with the emotional and existential support they are given. Even if they are satisfied with their medical and physical care, seriously ill patients often refrain from discussing their spiritual and existential thoughts with nurses because they do not feel that nurses acknowledge this need, in addition other studies show that nurses often feel unprepared to meet with patients facing spiritual and existential issues at the end of life, which can be a barrier against conducting spiritual and existential care for the dying [20–24]. According to Henoch and Danielson [25], there is a gap in the research literature about how patients’ existential wellbeing may be best supported by nurses and other health care professionals in everyday practice.

### Aim

The aim of this study is to describe nurses’ experiences with spiritual and existential care for dying patients in a general hospital.

The nursing literature interprets and applies the terms “spiritual” and “existential” care in different ways, which suggests that these terms are open to interpretation. Several scholars support this view [6, 23, 26–29] and according to them, there seems to be no single agreed definition on spiritual and/or existential care in nursing literature. In line with these scholars, this study has adopted a pragmatic and functionalist epistemological point of departure targeted at the practical implications of the nurses’ spiritual and existential care experiences rather than the ontological questions related to the conceptual framework. Based on our pragmatic point of view we have chosen to use the term “spiritual and existential care” consistently throughout this study.

## Method

### Design

This is a qualitative study. As such, its quest is not for objectivity and explanation as in natural sciences. Rather, the quest lays in seeking for the meanings and deeper understandings of the participants’ lived experience, and the subjective meanings of their experiences [30–32]. Henceforth, it is not reasonable to discuss the concepts of validity, reliability and generalizability in their traditional senses because the few informants chosen in qualitative research projects are not sufficient to allow conclusions to be generalized back to the population from which they were drawn. However, they do insure strength and representativity in relation to transferability, as they permit an in-depth insight into the phenomena under study. Qualitative projects can therefore be stated to show a high content validity. This means that there is a high degree of detail in the data. However, due to the lack of numerical representativity it is not possible to make inferences about the relative or absolute incidence of the observed phenomena in the background population [33].

The study was conducted using a qualitative phenomenological hermeneutical interpretation method developed by Lindseth and Norberg [34] which is inspired by Ricoeur’s phenomenological hermeneutical interpretation theory [31]. Lindseth and Norberg’s [34] interpretation method has proven to be suitable for illuminating lived experience in interview texts [34, 35] and has been used in several palliative care studies [15, 36–38].

According to Ricoeur [31], human action can be understood as discourse and interpreted as text, when it has been objectified and fixated through writing. When spoken discourses, such as research interviews, are written down, the texts (in this case the interview transcripts) become *“autonomous”* with respect to the interviewees’ intentions [31, 39, 40].

Ricoeur [31] points out that the fixated discourse and its meaning becomes distanced from the speech event. As such, the text becomes *“decontextualized”* from the speech event and its social and historical conditions. This opens up the text to an unlimited series of readings [31], where the readers will experience a need to recontextualize and *appropriate* the text, − to familiarize them selves with the text and make it their own.

Ricoeur [31] states that there exists a dialectic relationship between distanciation and appropriation in the interpretation process: *“To make one’s own what was previously foreign remains the ultimate aim of all hermeneutics….. This goal is achieved insofar as interpretation actualizes the meaning of the text for the present reader”* [31]. For Ricoeur [31], interpreting a text involves moving beyond understand what it says (its *sense*) to understanding what it talks about (its *reference*).

Geanellos [41] points out that Ricoeur’s interpretation theory has two stages: (i) explanation, − or what the text says and (ii) understanding, or what the text talks about. While explanation is directed toward analysis of the internal relations of the text (the parts) understanding is directed toward grasping the meanings the text discloses (the whole in relation to the parts) In this way, interpretive understanding goes forward in a continual movement between the parts and the whole allowing understanding to be enlarged and deepened [41]. *“Ultimately the correlation between explanation and understanding, between understanding and explanation is “the hermeneutic circle”* [39]. For Ricoeur [31], the sense of a text is not behind the text but in front of it. It is not something hidden, but something disclosed: *“What has to be understood is not the initial situation of discourse, but what points to a possible world….. The dimensions of this world are properly opened up and disclosed by the text”* ([31] p. 87–88).

Methodologically distanciation and appropriation allow researchers to move beyond the notion that only the research participants’ understanding is meaningful and or correct. It also allows the interpreters to interpret the same text faithfully, yet somewhat differently because it is acknowledged that texts have many meanings [41]. For Ricoeur [39], all interpretive activity involves a dialectic between guessing and validating. The interpreter must make an educated guess about the meaning of a part and check it against the whole and vice versa. Hence, guessing and validation are circularly related as subjective and objective approaches to the text [39]. Although there is always more than one interpretation, Ricoeur [39] emphasizes that all interpretations are not equal and that one must try to find the most probable interpretation.

### Participants

Six registered nurses, working in a combined medical and oncological ward in a general hospital in a rural Norwegian town, were interviewed. This ward had patients with palliative; as well a curative needs so the nurses were familiar with the challenge of providing spiritual and existential care for the dying as part of their daily work.

The head nurse was contacted to obtain permission to conduct the study. She was also asked to inform all of her nurses about the study and to distribute a formal request/ information sheet on behalf of the authors. The first six nurses who signed up for the interview participated in the study.

The inclusion criteria were that the nurses were interested in palliative care, had a variety of experiences and had spent time in this work. The participants were between the ages of 37 and 61 years, with nine to twenty one years of nursing experience. Four of the six participants had degrees in oncology nursing and palliative care.

### Ethical considerations

The study was conducted according to the Helsinki declaration [42, 43]. Approval was obtained from the Norwegian Social Science Data Service (NSD), project number 29973 and participants gave their written consent.

All of the participants had received a formal request/information sheet from the authors prior to the interviews. The information sheet described the study’s aim and background, emphasizing that participation was voluntary and that the participants were free to withdraw from the study at any given time, during or after the interview. It also explained that the interviews would be transcribed verbatim and that individual identifiers would be removed from the transcripts. The information sheet underlined that all audiotapes and interview transcripts would be stored in a password-protected computer and that the participants’ information would only be used to conduct this study and that all information would be deleted as soon as the study was completed. The information sheet also emphasized that the information would be deleted immediately if the participant wished to withdraw his or her consent at any given time.

The interviews took place in a secluded and quiet meeting room outside the ward, in order to avoid interruptions and to protect the participants’ confidentiality.

Before each interview the first author introduced herself and repeated the written information that the participants had received. She especially emphasized the measures that were taken to ensure the participants’ confidentiality and anonymity. She also explained her role as the researcher and what she expected from the participants and encouraged the participants to ask questions about the study and the interview procedure. Personal information about each participant was obtained and written down. The first author emphasized that this information would be kept under lock and key. Written consent was then obtained from each participant.

### Data collection

The interviews took place in August 2014 and were conducted in a hospital meeting room during the nurses’ working hours. The first author conducted the interviews, which lasted approximately one hour. She also audiotaped and transcribed the interviews verbatim. The interview strategy was designed as a narrative approach with one open-ended question, followed by clarifying questions when necessary:

*What are your experiences with providing spiritual and existential care to dying patients?*

This choice was based on the underlying presupposition, that the perspective of the interviewees is best revealed in narratives where they use their spontaneous language to talk about events [44, 45]. No definitions of spiritual and existential care were introduced at the commencement of the interviews because we wanted the participants to talk freely about what they considered as spiritual and existential care. The aim of the interviews was to obtain as many rich narratives as possible, minimally interrupting the nurses’ narrative flow and reflection by tying questions and comments to the informants’ responses and repeating their words whenever possible [46]. In addition, the first author followed up on the themes that the informants focused on in order to obtain the meanings of their narratives [47].

### Data analysis

Data was analyzed using Lindseth & Nordberg’s [34] phenomenological hermeneutical method for researching lived experience where each interview is looked upon as a text. In line with Ricoeur’s interpretation theory [31], Lindseth and Norberg’s interpretation method [34] implies a dialectic movement between the text as a whole and parts of the text and consists of three steps. The first step, which involved a *naïve reading*, consisted of an open-minded superficial reading where the text was reread several times to grasp the meaning of the text as a whole. The aim was to gain access to the nurses’ lived experience with spiritual and existential care. The naïve reading guided the *structural analysis*, which was the second step*.* The text was divided into meaning units that were condensed into themes and sub-themes. The objective of the structural analysis was to explain what the text was saying. The themes and sub-themes are presented in the results section. Finally, a *critical comprehension* was developed where the text was read as a whole, taking into account the authors’ preunderstanding, naïve reading, structural analysis, previous research and relevant theory [34, 35]. The critical comprehension is presented in the discussion section.

### Rigour

The interview provided a large amount of in-depth information about the meaning of the nurses’ lived experience. A text may have more than one possible interpretation [34, 35]. Henceforth, the interpretation presented in this study should be viewed as one of several possible ways to understand the nurses’ experiences. To ensure rigour all authors performed individual naïve readings, which were then discussed between the authors. This served as a guide for the first author who performed the structural analysis. To strengthen the credibility of the structural analysis, all authors critically reviewed and compared the first author’s results in light of their naïve readings. The authors conducted a meeting to discuss the interpretation of the results. We found them to be consistent with our naïve readings. This strengthened the trustworthiness of the study.

### Study limitations

Norway is becoming an increasingly pluralistic and multicultural society [48]. However, the study was conducted in a rural Norwegian town where the majority of the population and all of the informants were ethnic Norwegians coming mainly from a secularized or traditional Norwegian church background. Therefore the nurses had limited experiences with spiritual and existential care for patients from other ethnic backgrounds and/or religious traditions. Due to the study’s geographical and cultural context the study is limited to the nurses’ experiences with providing spiritual and existential care to ethnic Norwegian patients.

## Results

Three themes and six sub-themes from the structural analysis are shown in Table 1. Results will be presented in the text below, including quotes to illustrate the themes.

**Table 1:** Overview of themes and sub-themes that emerged in the interview text

Themes	Sub-themes
The elusive entanglement of Suffering	- *Uncovering spiritual and existential Suffering*
Spiritual and existential Care	- *Ambivalence and Uncertainty*
	- *Facilitating Reconciliation and Communication between Family members*
	- *Unburdening Patients to facilitate a peaceful Passing*
Conveying Consolation	- *Emotional Challenges*
	- *Emotional Containers*

### The elusive entanglement of suffering

The nurses experienced that patients’ spiritual and existential suffering emerged as subtle and somewhat elusive entanglements of physical, emotional, relational, spiritual and existential pain. The nurses related patients’ spiritual and existential suffering to their pain, struggle and distress concerning reconciliation with their past, their loved ones, and the fact that their time was running out. The nurses experienced that dying patients and their families could become lonely and alienated from each other when they were unable to share their burdens of fear, grief and sorrow. They saw that patients often were deeply anxious about how their children and spouses would cope without them. The nurses experienced that patients could express their anxiety of dying as an existential fear of disappearing *“into a black hole”*. They also encountered patients who experienced spiritual and existential suffering because they thought God had given them illness and pain to punish them for their sins.

#### Uncovering spiritual and existential Suffering

According to the nurses, spiritual and existential suffering (which did not always include religious aspects) could emerge spontaneously. This could for example happen during physical care or any other moment, especially when the nurses were able to convey that they had time to listen. The ability to zoom in on the fleeting moments, when patients wanted and needed to talk was therefore seen to be an essential skill.

Initiating and engaging in conversations about how patients experienced their situation proved to be an important means to alleviate their spiritual and existential suffering. Being able to pick up patients’ spiritual and existential suffering required *“a fine tuned antenna”*. The nurses emphasized that it was important to pay attention to the patients’ energy levels and emotional states, neither forcing nor avoiding spiritual or existential conversations. The nurses emphasized the necessity of establishing trust and rapport before they attempted to inquire about patients’ spiritual and existential needs. However once they had gotten to know the patients, some of the nurses expressed that they could be quite frank with them: *”I never ask about these things without obtaining permission.”* According to the nurse, she usually got permission when she asked for it:

*“Can I ask you a direct question? If the patient agrees I’ll ask questions like: How do you feel about dying? Have you spoken with your wife about your situation? - and so forth and so on.”*

### Spiritual and existential care

#### Ambivalence and uncertainty

The nurses had mixed feelings about providing spiritual and existential care, although they considered that it was important. The nurses said they hesitated to inquire about patients’ spiritual or existential needs due to lack of time, insufficient staff resources and the possibility of being interrupted. However, they also reflected that they sometimes used this as excuses to avoid the challenge of talking with patients about spiritual and existential concerns. The nurses did not consider themselves to be *“very religious”* or *“very Christian”* as they put it, and they felt that religion was a very private and personal matter. They were therefore uncertain about how to address patients’ spiritual concerns, without imposing themselves on them. In spite of their ambivalences, our results show that the nurses attempted to provide spiritual and existential care within their limited time and resources. The nurses felt that alleviating spiritual and existential suffering was a complex and challenging task, depending on the patients’ family situation, social network, physical and mental condition, spiritual, religious and existential beliefs and their ability to communicate.

#### Facilitating reconciliation and communication between family members

Helping patients and families to achieve reconciliation was an important part of the nurses’ spiritual and existential care. They placed great emphasis on encouraging family members to talk with each other in order to share their grief, make their peace and say their goodbyes. The nurses saw that patients occasionally tended to conceal their impending death because they wanted to spare their loved ones. The nurses tried to urge these patients to talk openly with their families:

*“You’re really not sparing them you see, because they sense that something is wrong and they worry and wonder about what’s the matter with you! And besides, they want to help you if you’ll let them!”*

The nurses offered to initiate and moderate family meetings when they saw that families struggled to talk:

*“Occasionally some patients and families need a little persuasion to talk with each other. Sometimes we can provide that. But you have to tread carefully!”*

When the nurses saw that a divorced single father spent all his energy worrying about how his teenage son would manage without him, they asked the patient if he wanted help to talk with his son and ex wife: ”*He became so touched and relieved that he started to cry!”* The patient became calmer after the family meeting because he knew that his ex wife would take care of their son. The nurses frequently experienced that patients could become quite relieved when they were given the opportunity to express their feelings and talk about their worries and concerns. However, the nurses also experienced that some patients did not wish to talk with them or anybody else, and they reflected that it was important to respect the patients’ wishes.

#### Unburdening patients to facilitate a peaceful passing

The results show that several kinds of issues and concerns could exacerbate patients’ spiritual and existential suffering. It was therefore vital to uncover these issues to provide relevant interventions. This is especially illustrated in the nurse’s narrative about a young woman who was dying of lung cancer. Sensing her anxiety, the nurse asked her what she thought about death. The patient feared that she would just disappear into *“a big black hole”* and asked what the nurse thought about death: *”Isn’t there anything more afterwards?”* The nurse tried to relieve her anxiety by sharing her own hope of reuniting with her loved ones: *“I think they will be there to greet and welcome me when I die”.* This seemed to kindle the patient’s hope of reuniting with her diseased father: *“Maybe he’s standing there waiting for me!”* she exclaimed*.*

Although she seemed less anxious, the nurse observed that the patient was still agitated and distressed because she could no longer take charge of renovating the family home. The nurse recognized that the patient would not find peace until this was taken care of. She therefore contacted the social worker, who organized the patient’s family and friends to finish the home:

*“She still worried about her family because she was going to die from her kids. So in this case, unburdening her with the practical stuff was an important part of spiritual care.”*

The nurse relieved some of the patient’s death anxiety by sparking her hope of reconnecting with her diseased father. By intervening to fix her house, the nurse helped the patient to maintain her family role and responsibilities the rest of her life.

The complexity of identifying and alleviating spiritual and existential suffering was also elucidated in a narrative about a patient who believed that God was using her illness and pain to punish her. In this case the nurse had to deal with the patients’ religious issues before she could relieve her physical pain. Desperately searching for a way to reach in to the patient, it suddenly dawned on her that she could use prayer *“to turn the situation around”.* So she asked if the patient wanted to say the “The Lords Prayer” with her. According to the nurse, sharing the prayer helped her to connect with the patient. This opened up a natural opportunity to talk with her about her picture of a punishing and vengeful god:

“*That was the first time I had been so frank with her about religion. I sat by her bedside after the prayer, and we talked a lot about why she thought God was using cancer and pain to punish her.”*

Although the nurse claimed she was not *“very religious”*, she shared her belief in a trusting and loving God:

*“The God I believe in loves us and wants to help us! And now I can help you to take your pain away, − at least some of it! - If you’ll let me!”*

By praying with the patient and addressing her religious distress, the nurse managed to obtain the patient’s permission to alleviate her physical pain. The nurse reflected that it was only after they had prayed together that the patient’s attitude changed:

“*I really believe that when she experienced that I took her spiritual pain seriously, she also became willing to let me alleviate her physical pain.”*

After that the patient allowed the nurses to give her morphine regularly. According to the nurse, the patient died pain free and at peace with her God!

### Conveying consolation

The nurses’ narratives tended to evolve around their efforts to help their patients achieve peace and tranquility. The nurses strove to help patients accept death, settle their practical affairs and achieve reconciliation with their past, their loved ones, and with God. The nurses felt that they had been able to convey consolation when they saw that their efforts had helped patients to experience a good, peaceful and harmonious death. This was experienced as very rewarding and fulfilling. Bearing witness to the peaceful passing of a patient was described as a special moment that filled them with reverence and awe:

“*The room was very quiet and the patient died calmly and peacefully. It was a very special moment.”*

The nurses could become deeply touched and amazed when patients shared their trust and openness:

*“Sometimes I’m really astonished, that they choose to share their troubles and worries with me! Even though I’m their nurse, I ‘m still a stranger!”*

The nurses said they felt honored when patients chose to confide in them.

#### *Emotional challenges*

However, being the patients’ confidant could also be emotionally draining:

*“I can become very overwhelmed when patients share their innermost thoughts and feelings about life and death! It almost knocks me out sometimes!”*

Bearing witness to younger patients deaths could make the nurses feel vulnerable, especially when they identified with their patients:

*“One of my patients had a little baby. That was really tough, because I am a mother myself!”*

The nurses experienced that their greatest emotional challenge was to endure being with patients who continued to radiate anguish, protest and denial in spite of the nurses’ efforts to console them. These encounters activated their feelings of professional helplessness and inadequacy:

*“A young cancer patient anxiously battled death till the bitter end. All of us thought it was terrible, the way he died! We really tried, but nobody could help him find peace because****he simply refused to die****! We sat there holding his hand and listening to him. But he was completely inconsolable! It was very, very challenging and frustrating, even though we know that we probably did all we could!”*

The nurses reflected that their reactions stemmed from the need to see results from their work and to be able to feel that they were good nurses:

“*I guess we react like this, because it’s more rewarding to help patients and their families to find peace than it is to cope with angry patients who protest and refuse to accept their situation”.*

Occasionally patients and families threw their anger and frustration at the nurses: *“We sometimes get a lot of verbal abuse that can seem extremely unfair”.* Although the nurses understood that patients and family members vented their pain and suffering by taking it out on them, they still experienced that this could be emotionally challenging. Nevertheless, the nurses emphasized that they were obliged to alleviate the patient’s and the family’s distress as best they could:

“*Then we have to stop and think: It’s usually neither their nor our fault. We have to remember that they are in a very difficult situation. After all they are struggling with the existential questions of life and death. So of course it’s difficult for them!”*

The results reveal that the nurses yearned to convey consolation and found it difficult to accept that this was not always possible: “*As nurses, we’re very into problem solving and we really want to “fix” the patients’ problems.”* On an emotional level, the nurses tended to feel that they had not done a good enough job when they experienced that they were unable to help their patients find peace and reconciliation in the final stages of dying: *“It’s very hard for us to accept that we can’t solve people’s suffering!”* However, on a rational level, the nurses acknowledged that it was necessary to accept that they could not console everybody. Drawing on this insight, the nurses emphasized that conveying consolation by just being willing to be there, to share the patients’ suffering was more important than trying to resolve the patients’ spiritual and existential distress:

“*We can’t solve everybody’s problems. There is such a thing as pointless suffering! We have to accept that things don’t always have a deeper meaning.”*

#### *Emotional containers*

The results show that the nurses had an important function as “emotional containers” when they listened and encouraged patients and families to vent their thoughts and feelings:

*“It doesn’t do any harm if people start to cry. I usually tell my patients that they don’t have to feel ashamed of their tears. Tears are only melting ice! ”*

However, the results also show that nurses could be reluctant to take on this containing function because the patient’s distress exposed them to their own anxiety related to suffering and dying. In the nurses’ experience, older nurses seemed to be more willing to engage in the patients’ spiritual and existential distress than their younger colleagues. They assumed that the older nurses' personal and professional life experiences had made them more robust to bear the weight of the patients’ distress than the younger nurses. The nurses emphasized that conveying consolation and containing other people’s emotions demanded personal courage, maturity, and a will to be touched by the patients’ tears and emotions:

“*You have to come to terms with your own thoughts and feelings about your own vulnerability to endure working here over time. It’s a demanding job! Not all nurses are cut out to care for the dying!”*

In the nurses’ experience, what patients needed most were nurses who were willing to endure and stand by their patients showing that they would not abandon them in their time of need:

“*When I saw that her time was running out, I told my colleague that I was going to stay with her. I just sat there holding her hand, and occasionally stroked her hair and her forehead. Sometimes I murmered a few words to let her know that she wasn’t alone.”*

## Discussion

In this study the nurses narrated about their experiences with spiritual and existential care for dying patients in a medical and oncological ward in a general hospital. Three themes emerged through the interpretation of the results: Becoming ready for consolation, Non-doing presence, and Finding the zone of middle engagement.

According to the results, the nurses experienced that patients’ spiritual and existential suffering emerged as subtle and elusive entanglements of physical, emotional, relational, spiritual and existential pain. The nurses saw that patients could need help to alleviate death anxiety, achieve peace and reconciliation with God and/or family, settle practical affairs and unfinished business, set their homes in order, and resolve family conflicts and worries. These results are supported by Steinhauser et al. [4] who found that resolutions within the biomedical, psychosocial or spiritual domains of patients’ experiences often proceeded their subjective experiences of peacefulness and that peacefulness was often related to an antecedent broader theme of “completion” or life closure.

Some of the results will be discussed in light of the work of Cassel [49] and Norberg et al. [50] who state that suffering can be understood as a kind of alienation and a threat against a person’s sense of identity, integrity and connectedness. According to Norberg et al. [50] consolation is needed when a human being feels alienated from him or herself, from other people, from the world and from his or her ultimate source of meaning. Furthermore, Norberg et al. [50] point out that consolation can be understood as a form of healing that involves a changed perception of the world in suffering persons. This healing shift of perception enables suffering patients to set their suffering within a new pattern of meaning, in a new transcendent light. Quoting the existential philosopher Søren Kierkegaard, Norberg et al. [50] declare that:

“*it is in the fearful moment of desolation, when there is no meaning left, that a brave statement of consolation penetrates the darkness and creates new meaning. This happens on the boarder where nothing is possible anymore.”*

This is illustrated in the nurse’s narrative about the young woman who feared that she would disappear into a black hole when she died. By sparking the patient’s hope of reconnecting with her loved ones, the nurse managed to help the patient to shift her perspective of death. This enabled her to transcend her dark fear of slipping into the black hole of oblivion.

In the narrative about the patient that thought God was using her illness to punish her, the nurse was able to help the patient change her perception of a wrathful and avenging God to a loving God that cared for her. In both of these narratives the nurses were able to convey consolation by helping their patients to transcend the isolating loneliness of spiritual and existential suffering.

The results show that the nurses experienced that they had been able to convey consolation when they observed transformations in the patients’ demeanor, − shifting from states of physical and emotional restlessness, pain and anxiety, towards states of peacefulness and tranquility. The nurses observed that such changes took place when they had been able to unburden some of the patients’ most pressing sources of anxiety and distress. The nurses’ emphasis on helping their patients to achieve peace, reconciliation and harmony in the final stages of dying can be understood in light of the early hospice movement’s *“good death ideology”* where open communication, relief of symptoms, individual dignity and respect and acceptance of death are prominent features [1]. According to Costello [1], the hospice movement’s *“good death ideology”* has been sustained from traditional times to post-modern society and still permeates many aspects of contemporary palliative care. Costello [1] points out that *“good deaths”* are often sentimentally idealized as being personal and individualized, evoking images of death as peaceful, natural and dignified. A number of studies indicate that the fewer difficulties patients experience in their passage towards death, the greater the likelihood of the death being positively perceived by the nurses, whereas *“bad deaths”* had the potential to cause trauma and a sense of crisis for dying people and others. *“Bad death”* experiences were often referred to as *“traumatic, chaotic or gruesome”* by the nurses ([1] p. 595).

As mentioned earlier, our results show that conveying consolation in the final stages of dying could be an emotionally challenging and complicated endeavor, which was particularly revealed in the narrative about the young cancer patient who protested and fought against death until the bitter end. The nurses’ reactions are understandable in light of the hospice movements *“good death ideology”.* Palliative care practice has been heavily influenced by this ideology and maintains a normative and strong emphasis on the significance of wellbeing and a “good ending” which involves diminishing anxiety and facilitating a sounder grieving process, for patients as well as their families, and of staying connected and reconnecting with “lost” relationships [1, 51]. Taking this into consideration, it seems reasonable to believe that the nurses considered the dying young man’s resistance as a *“bad death experience”*:

*“All of us thought it was terrible the way he died! He was completely inconsolable! It was very, very challenging and frustrating! – even though we know that we probably did all we could!”*

Torjuul et al. [52] point out that suffering is not a morally neutral phenomenon. Rather it is perceived and judged as something that should not be there and awakens the immediate response in nurses to alleviate it, ameliorate it and prevent it whenever possible:

*“The ultimate purpose for health care personnel is to combat and alleviate suffering and that clients and families should experience as little suffering as possible”* ([52] p.529).

In light of this ultimate purpose it is understandable that the nurses yearned to convey consolation in order to help their patients to achieve a peaceful, uncomplicated and harmonious death. However, according to Norberg et al’s [50] consolation model both nurses and their patients must become ready for consolation. Nurses become ready to convey consolation through their willingness to see and listen to the suffering patient. The patients must reach a state of openness where they are willing to endure the pain of exposing their feelings of desolation and despair.

Our results imply that some patients may never reach this state of openness because they are unable or unwilling to surrender their protective shield and face the pain related to their impending death. This poses an ethical challenge for the nurses. In their eagerness to convey consolation, nurses are at risk of violating their patients’ autonomy due to the asymmetrical nature of the nurse-patient relationship. Although Norberg et at [50] state that it is beneficial for patients to *“uncover and look at their wounds”*, patients’ resistance to do so can also be understood as a coping strategy they need and choose:

*“While the truth is desirable, denial is not necessarily a negative mechanism, but can be a gradual means of coming to terms with the situation. It is a healthy reaction, allowing the person time to adapt and later draw on defense mechanisms*” ([53] p.208) quoted in Zimmerman [54]*.*

It is reasonable to assume that nurses may be at risk of tearing off a much-needed protective scab of denial if they are too forthright in their questioning. The nurses in our study were concerned about obtained permission before they asked sensitive questions related to the patients’ thoughts and feelings about their impending death. However, given the asymmetrical power structure in the nurse-patient relationship it is questionable whether or not vulnerable patients are in a position to deny their nurses such permission. Henceforth, a major challenge related to conveying consolation, is to resist the temptation of imposing well-meant interventions on patients. The nurses must therefore be able and willing to *“walk alongside the patient”* [50]. Although sharing the patients’ suffering in a *“presencing and non-doing fashion”* may be emotionally challenging, nurses must put aside their personal needs to console patients who are not ready to receive consolation.

This is in line with Back et al’s [8] research. They point out that the danger of being unaware of feeling helpless or being unwilling to experience that one feels helpless may bias the clinician’s attention. Clinicians in the grip of helplessness are likely to proceed with a constricted view of the patient’s situation meaning that important facts will be missed and conclusions drawn prematurely. As a consequence the patient’s views, values and stories will be insufficiently seen and may distort palliative care into a series of medical treatments and procedures.

In order to sustain their ability to convey consolation in a non-doing, non-invasive manner Back et al. [8] suggest that clinicians must learn to reframe their experience of helplessness from one of *“being at the end of the road to being in a moment of relationality with the patient”* , and they argue that re-experiencing helplessness as a moment in a clinician-patient relationship can enable the clinician to shift to a middle place between a "hypo-active engagement" (characterized as a resigned, passive and apathetic state) and a "hyper- active engagement" (characterized by an anxious, pressured, vigilant, even desperate state). Back et al. [8] hypothesize that there exists “*a middle zone of engagement”* which they describe as the *“zone of constructive engagement”*.

Working in this zone clinicians are willing to put in a cognitive, emotional and spiritual effort that goes beyond the guidelines of professional competence. When clinicians are working from their constructive zone they are able to evaluate the situation for what it is, empathizing without getting overwhelmed, drawing on their wisdom and expertise while at the same time experiencing moments of effectiveness and moments of disappointment. According to Back et al. [8] *“the zone of constructive engagement”* is not a static state, but a range of possible experiences that reflects the dynamic nature of clinical work. Back et al. [8] state that reframing helplessness enables clinicians to reframe vulnerability from being *“a soft underbelly”* that must be hidden and protected to an essential connection with the tragedy and fragility of being human. This is in line with our results. As one of the nurses put it:

“*You have to come to terms with your own thoughts and feelings about your own vulnerability to endure working here over time. It’s a demanding job! Not all nurses are cut out to care for the dying!”*

The results in our study show that the nurses fluctuated between all three states of "hypo", "hyper" and "constructive engagement". The following quote can be interpreted as an experience of "hypo-engagement":

*“I can become very overwhelmed when patients share their innermost thoughts and feelings about life and death! It almost knocks me out sometimes!”*

On the other hand, the nurses’ needs to see results from their work and to feel that they were good nurses could drive them into a state of “hyper-engagement":

“*As nurses we’re very into problem solving and we really want to “fix” the patients’ problems”.*

Back et al. [8] point out that how nurses respond to their own helplessness is likely to shape the suffering of their patients. Although the nurses expressed ambivalence, the results also show that they had the courage and willingness to enter into the middle zone of constructive engagement when they functioned as *“emotional containers”* listening and encouraging their patients and families to vent their thoughts and feelings.

Our results show that the challenge of consolation is related to the nurses’ unavoidable experience of helplessness in the presence of dying patients’ existential and spiritual suffering. Yet human beings are not only passive perceivers in the context of social interactions. Back et al. [8] point out that human beings are also active creators of shared emotional experiences in line with Norberg et al’s [50] work. When the nurse and the patient become ready to give and receive consolation at the same time, they are in a state of communion where mutual consolation may take place. The patient may draw consolation from the nurses’ presence and the nurse may draw consolation when he or she experiences that the patient is able to move from a state of anguish, suffering and distress towards a state of peacefulness and tranquility [50].

## Conclusion

Although spiritual and existential suffering at the end of life cannot be totally alleviated, nurses may ease some of the existential and spiritual loneliness of dying by standing with their patients in their suffering. Further research (qualitative as well as quantitative) is needed to uncover how nurses provide spiritual and existential care for the dying in everyday practice. Such research is an important and valuable knowledge supplement to theoretical studies in this field.
